# Cytomegalovirus results in poor graft function via bone marrow-derived endothelial progenitor cells

**DOI:** 10.3389/fmicb.2024.1463335

**Published:** 2024-09-18

**Authors:** Weiran Lv, Ya Zhou, Ke Zhao, Li Xuan, Fen Huang, Zhiping Fan, Yuan Chang, Zhengshan Yi, Hua Jin, Yang Liang, Qifa Liu

**Affiliations:** ^1^Department of Hematologic Oncology, State Key Laboratory of Oncology in South China, Guangdong Provincial Clinical Research Center for Cancer, Sun Yat-sen University Cancer Center, Guangzhou, China; ^2^Department of Hematology, Nanfang Hospital, Southern Medical University, Guangzhou, China

**Keywords:** poor graft function, human cytomegalovirus, endothelial progenitor cells, transforming growth factor beta, vitamin D receptor

## Abstract

**Introduction:**

Poor graft function (PGF), characterized by myelosuppression, represents a significant challenge following allogeneic hematopoietic stem cell transplantation (allo-HSCT) with human cytomegalovirus (HCMV) being established as a risk factor for PGF. However, the underlying mechanism remains unclear. Bone marrow endothelial progenitor cells (BM-EPCs) play an important role in supporting hematopoiesis and their dysfunction contributes to PGF development. We aim to explore the effects of CMV on BM-EPCs and its underlying mechanism.

**Methods:**

We investigated the compromised functionality of EPCs derived from individuals diagnosed with HCMV viremia accompanied by PGF, as well as after infected by HCMV AD 169 strain *in vitro*, characterized by decreased cell proliferation, tube formation, migration and hematopoietic support, and increased apoptosis and secretion of TGF-β1.

**Results:**

We demonstrated that HCMV-induced TGF-β1 secretion by BM-EPCs played a dominant role in hematopoiesis suppression *in vitro* experiment. Moreover, HCMV down-regulates Vitamin D receptor (VDR) and subsequently activates p38 MAPK pathway to promote TGF-β1 secretion by BM-EPCs.

**Discussion:**

HCMV could infect BM-EPCs and lead to their dysfunction. The secretion of TGF-β1 by BM-EPCs is enhanced by CMV through the activation of p38 MAPK via a VDR-dependent mechanism, ultimately leading to compromised support for hematopoietic progenitors by BM EPCs, which May significantly contribute to the pathogenesis of PGF following allo-HSCT and provide innovative therapeutic strategies targeting PGF.

## 1 Introduction

Allogeneic stem cell transplantation (allo-HSCT) is a well-established therapeutic modality aimed at achieving a cure for individuals diagnosed with hematological malignancies or non-malignant disorders (Liu et al., 2014; Shi et al., 2016). Poor graft function (PGF) constitutes a significant adverse outcome subsequent to allo-HSCT with poor prognosis characterized by severe cell depletion in at least two lines of erythroid, granuloid and megakaryocyte (Liu et al., 2014; Shi et al., 2016). Nevertheless, the etiology of PGF remains obscure and the therapeutic effect is not satisfactory. Therefore, exploring the pathogenesis of PGF can contribute to the identification of potential novel targets and strategies for the prevention and therapy of PGF, so as to effectively improve the survival rate after allo-HSCT.

Cytomegalovirus (CMV) is the most common viral infection in allo-HSCT recipients. Relevant clinical studies have proved that CMV plays a significant role in the occurrence PGF (Nakamae et al., 2011; Lv et al., 2021) and results in abnormity of hematopoietic reconstitution post-transplant (Movassagh et al., 1996; Hancock et al., 2020). CMV can cause hemopoietic inhibition by infecting bone marrow stem progenitor cells and bone marrow stromal cells (Kumar and Geiger, 2017; Ding et al., 2012; Goodrum et al., 2004). Hematopoiesis is intricately regulated by a sophisticated interplay between hematopoietic and stromal cells, as well as a multitude of soluble and membrane-bound cytokines within both the intra- and extra-hematopoietic niches (Giudice et al., 2021). Bone marrow-derived endothelial progenitor cells (BM-EPCs) play a pivotal role as essential cellular components within the stromal cells residing in the bone marrow (BM) microenvironment, exerting a significant influence on supporting hematopoiesis (Ding et al., 2012; Calvi and Link, 2015). New findings from studies conducted on mice and humans suggest that the reduced number and abnormality of EPCs, characterized by compromised capacities in cellular proliferation, migration, and angiogenesis, alongside elevated levels of apoptosis, are linked to development of PGF (Kong et al., 2013; Salter et al., 2009). Furthermore, BM-EPCs possess the capacity to secrete an array of hematopoietic-related cytokines which could potentially contribute significantly to the regulation of hematopoietic processes (Fennie et al., 1995; Maki et al., 2018). Nevertheless, there is currently limited knowledge regarding the impact of HCMV on BM-EPCs, particularly on their ability to support hematopoiesis.

It has been suggested that there is an association between dysregulation in vitamin D metabolism and a significant impact on the outcome of HSCT, especially caused by inadequate levels of vitamin D (Zhu et al., 2021). In addition to its classical role in maintaining calcium and phosphorus homeostasis, vitamin D is increasingly acknowledged for its pivotal involvement in immune regulation and antiviral defense mechanisms (Stecher et al., 2022). The role of Vitamin D receptor (VDR) is pivotal in mediating the diverse effects of vitamin D. The transcriptional activity of VDR is further augmented by the binding of calcitriol, the biologically active form of vitamin D (Ryan et al., 2015). Emerging evidence has revealed that HCMV infection is associated with the down-regulation of VRD expression, while reduced VRD levels are not consistently observed in other viral infections (Rieder et al., 2017). A general reduction in VDR levels has been observed in hematopoietic stem cell transplant patients with active HCMV infection (Robak et al., 2021). Previous studies have revealed that the upregulation of VDR levels significantly enhances the viability, proliferation, and migration of EPCs while reducing their apoptosis (Xiang et al., 2016; Grundmann et al., 2012). However, no previous studies have focused on the roles of VDR on BM-derived EPCs during CMV infection.

Mitogen-activated protein kinases (MAPKs), a group of enzymes belonging to the serine/threonine kinase family, comprises three primary subgroups: extracellular signal-regulated kinase (ERK), p38 MAPK (p38), and JNK (Shi et al., 2016). The impaired functionality in cultured EPCs in PGF patients has been reported to be significantly regulated by the crucial involvement of p38 MAPK (Shi et al., 2016), which indicates the significant involvement of MAPKs in development of PGF. Moreover, Zhu *et al* reported that Vitamin D3 mitigates pulmonary fibrosis by modulating the MAPK pathway in both *in vivo* and *in vitro* settings (Zhu et al., 2021). However, the participation of the MAPK pathway in the regulation of the hematopoietic related dysfunction of BM-EPCs after HCMV infection remains unknown.

We propose that the pathogenesis of HCMV-induced PGF involves modulation of BM-EPCs. To verify this hypothesis, our study investigated the impact of HCMV on BM-EPCs and elucidated the underlying mechanism.

## 2 Materials and methods

### 2.1 Viruses

The generous contribution of Human Embryo Fibroblasts (HF) and Recombinant HCMV Strain AD169 was provided by Professor Guojun Wu (Xiangya Medical College of Central South University). A single stock of AD169 virus at 3 × 107 plaque-forming units (PFUs)/ml was used throughout the subsequent experiment.

### 2.2 Isolation, cultivation, and identification of BM-EPCs

Isolation, cultivation and identification of BM-EPCs were according to literature (Shi et al., 2016). Briefly, the BM mononuclear cells (MNCs) were separated using density gradient centrifugation, a technique employed for cell separation. Subsequently, they were cultured with EGM-2-MV-SingleQuots from Lonza in Walkersville, MD for a duration of 7 days prior to undergoing testing. After 1 week of cultivation, the BM adherent cells were identified as EPCs through the utilization of Flow cytometry to detect CD34, CD133, and vascular endothelial growth factor receptor 2 (CD309). Dil-Acetylated Low Density Lipoprotein (DiI-AcLDL) uptake and fluorescein isothiocyanate–labeled *Ulex Europaeus* Agglutinin-I (FITC-UEA-I) binding assay were also used to identify the characterization of EPCs and assess the quantities of EPCs exhibiting dual positive staining as previously described (Shi et al., 2016). At day 7 of cultivation, EPCs were characterized by the expression of CD34, CD309, and CD133 and their capacity to uptake DiI-AcLDL and bind FITC-UEA-I.

### 2.3 HCMV infects EPCs and HCMV detection

Isolation, cultivation and identification of BM-EPCs were according to literature (Shi et al., 2016). For infection *in vitro*, the adherent EPCs from healthy donors of cultivation at 1–2 passage were exposed to HCMV AD169 at a multiplicity of infection (MOI) of 5 PFUs per cell for a duration of 2 h. Following infection, phosphate-buffered saline (PBS) was employeed to rinse cells in order to eliminate any remaining unabsorbed virus. Continuous cultures were established for 96 h, and EPCs were collected for the following experiments. EPCs from health donors which were not infected by HCMV were used as normal controls.

Indirect immunofluorescence (IF) was performed to detect HCMV pp65 in EPCs. HCMV-DNA of plasma in human subjects was quantified using quantitative real-time polymerase chain reaction (qRT-PCR), and the threshold for HCMV-DNA was < 500 copies/ml (Xuan et al., 2012).

### 2.4 Assessment of cell proliferation, apoptosis, and angiogenesis of BM-EPCs

VDR agonist (Calcitriol (1,25(OH)_2_D_3_)) and p38 inhibitor (SB203580) were purchased from Sigma (USA). After treatment with Calcitriol (50nM) and SB203580 (10uM), the adherent EPCs in culture were rinsed using PBS and gently released using trypsin supplemented with 0.125% EDTA. The analysis of BM-EPCs encompassed assessment of cellular proliferation, evaluation of migratory capacity, examination of tube formation potential, and investigation of apoptosis, was performed in accordance to the previous investigation (Shi et al., 2016).

### 2.5 Coculture of BM CD34+ cells with EPCs

A CD34 MicroBead Kit was used to isolated BM CD34+ HSCs from healthy donors. The isolated CD34+ cells were cultured in StemSpan SFEM without direct contact and separated from adherent EPCs using 0.4-mm transwell inserts for a duration of 4 days. The hematopoietic ability of BM CD34+ cells was determined by analyzing enumeration of colony-forming units (CFUs) following co-culture with MethoCult H4434 Classic (Stem Cell Technologies) and 24-well plates were used to culture cells at a density of 2 × 103 for a duration of 14 days. The evaluation of colony-forming unit erythroid (CFU-E), burst-forming unit erythroid (BFU-E), colony-forming unit granulocyte-macrophage (CFU-GM), and colony-forming unit granulocyte, erythroid, macrophage, and megakaryocyte (CFU-GEMM) measurements were conducted using an inverted light microscope (Supplementary Figure S1). To counteract the effects of TGF-β, supernatants were treated with an anti-TGF-β antibody (clone 1D11, MAB1835, R&D) at a concentration of 1 ug/mL before their combination with CD34+ BM cells and coating for colony assessments as previously describe.

### 2.6 Western blotting

EPCs were collected and disrupted in a lysis buffer (RIPA) supplemented with proteinase inhibitor and PhosSTOP (Fabio science, China). BCA protein assay kit (Fabio science, China) was used to detect protein concentration. The primary antibodies utilized in this investigation were mouse monoclonal antibodies (McAbs) that specifically targeted glyceraldehyde-3-phosphate dehydrogenase (GAPDH), phosphorylated forms of p38 MAPK, ERK and JNK as well as the total protein levels of p38, ERK and JNK (Cell Signaling Technology). The dilutions of these antibodies were prepared following the manufacturer’s recommended protocols.

### 2.7 qRT-PCR

The extraction of total RNA was performed utilizing the TRIZOL reagent (Takara). The mRNA levels of hematopoietic cytokines and GAPDH were was performed by SYBR green-based RT-qPCR using a *Roche*LightCycler480 (Roche Molecular Systems, Inc. America). The primers were shown in the Supplementary Table S1.

### 2.8 Enzyme-linked immunosorbent assay (ELISA)

The BM-EPCs with HCMV infection (HCMV Infected group) and without HCMV infection (Normal control group) were cultured in media without serum for an additional 96 h before collecting the supernatants containing secretome samples (secretome samples). Secretome samples obtained in the absence of cellular presence from BM-EPCs in HCMV Infected group or Normal control group were analyzed for secreted TGF-β1 by ELISA kit (R&D Systems, Minneapolis, MN, United States) following the manufacturer’s recommended protocols.

### 2.9 Samples from human subjects

BM sample was obtained from patients with HCMV viremia (HCMV-emia) accompanied by PGF without antiviral treatment (HCMV(+) + PGF group), patients without HCMV-emia accompanied by PGF without antiviral treatment (HCMV(−) + PGF group), patients without HCMV-emia and good graft function (HCMV(−) + GGF group), patients with HCMV-emia and good graft function (HCMV(+) + GGF group). The patients in above four groups were selected from a shared cohort, ensuring that age, pretransplant conditions, and post-transplant duration were appropriately matched. The four groups did not exhibit any notable distinctions with regards to clinical features, including primary malignancies, CD34+ cell dose transfused for transplantation, and previous episodes of acute graft-versus-host disease (GVHD) (Supplementary Table S2). Healthy individuals were recruited as control subjects (donor group). All subjects were obtained with consent. The present study obtained ethical approval from the Ethics Committee at Nanfang Hospital. The definition of PGF (Liu et al., 2014; Shi et al., 2016; Sun et al., 2019) is the presence of 2–3 consecutive instances where blood cell counts fall below normal levels (neutrophils ≤ 0.5 × 109/L, platelets ≤ 20 × 109/L, and/or hemoglobin ≤ 70g/L) for a minimum duration of three days after day + 28 following HSCT or when there is a dependence on transfusions; the presence of hypoplastic-aplastic bone marrow (BM) in cases exhibiting complete donor chimerism; and the absence of concurrent active GVHD or malignancies relapse. The good graft function (GGF) (Liu et al., 2014; Shi et al., 2016; Sun et al., 2019) exhibited an ANC of less than 0.5 × 109 /L for a continuous period of 3 days, PLT count below 20 × 109 /L for seven consecutive days, and Hb level lower than 70g/L without requiring any transfusion support beyond day 128 post-HSCT. HCMV viremia was defined as HCMV-DNA ≥ 500 copies/ml in plasma measured by qRT-PCR (Xuan et al., 2012).

### 2.10 Statistical analysis

**Figure 1:**
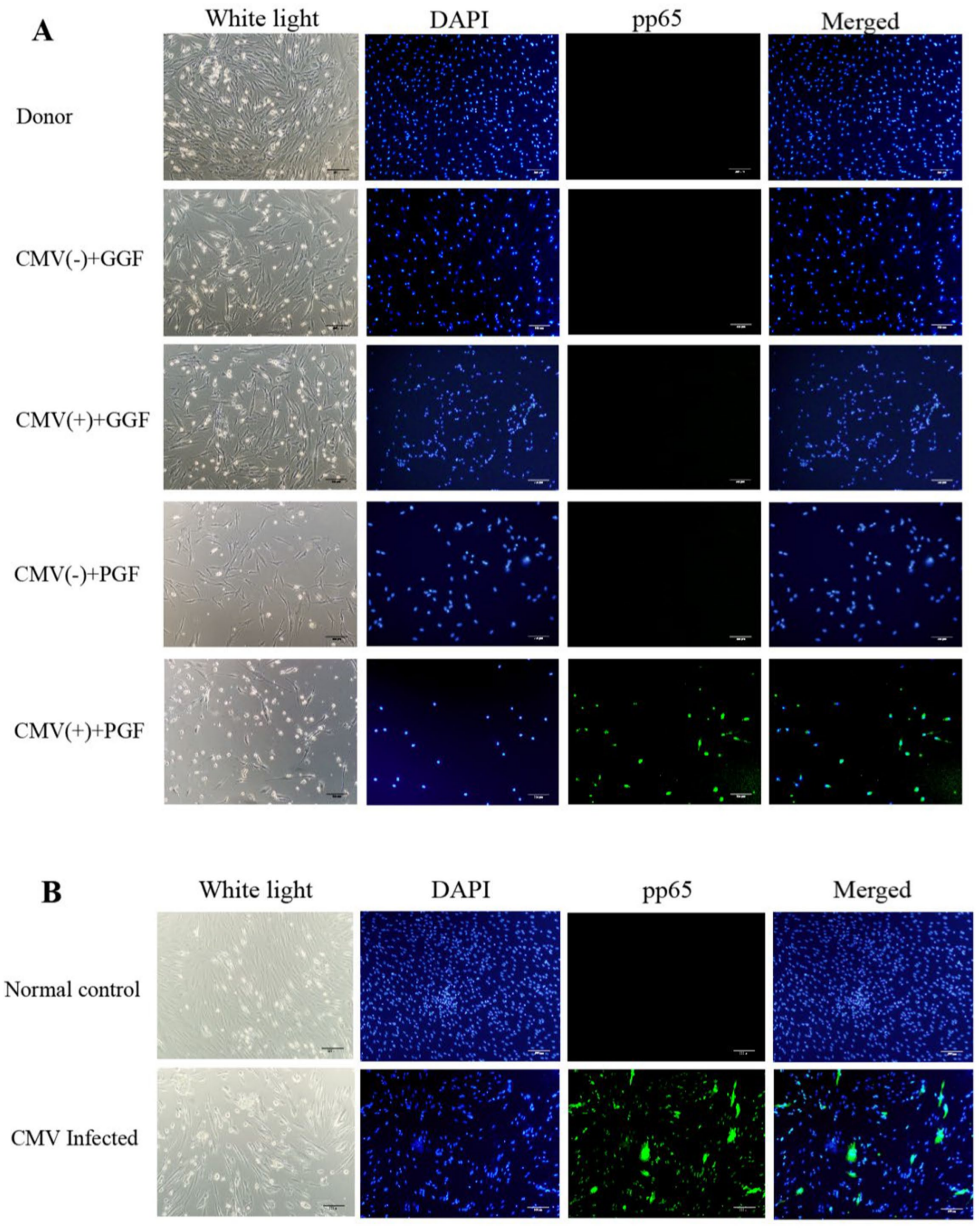
Infection of BM-EPCs with CMV. **(A)** Detection of CMV infection in primary BM-EPCs drived from patients after allo-HSCT. Cultivated BM-EPCs at day 7 in culture among subjects with donor group, CMV(−) + GGF group, CMV(+) + GGF group, CMV(−) + PGF group and CMV(+) + PGF group were observed through an inverted microscope. Active CMV infection was characterized by expression of CMV pp65 protein (green) in the nuclear through an inverted fluorescent microscope (scale bars, 200um, original magnification ×10). **(B)** For infection *in vitro*, the adherent BM-EPCs from healthy donors of cultivation at 1–2 passage were exposed to HCMV AD169 at a multiplicity of infection (MOI) of 5 PFUs per cell for 2 h. Following infection, cells were washed in phosphate-buffered saline (PBS) to remove unabsorbed virus. Continuous cultures were established for 96 h, and BM-EPCs were collected for the following experiments. The adherent BM-EPCs from healthy donors of cultivation at 1–2 passage were exposed to HCMV AD169 (CMV infected group) and not exposed to HCMV AD169 (normal control group). Both groups were observed through an inverted microscope. Active CMV infection was characterized by expression of CMV pp65 protein (green) in the nuclear through an inverted fluorescent microscope (scale bars, 200um, original magnification ×10).

**Figure 2:**
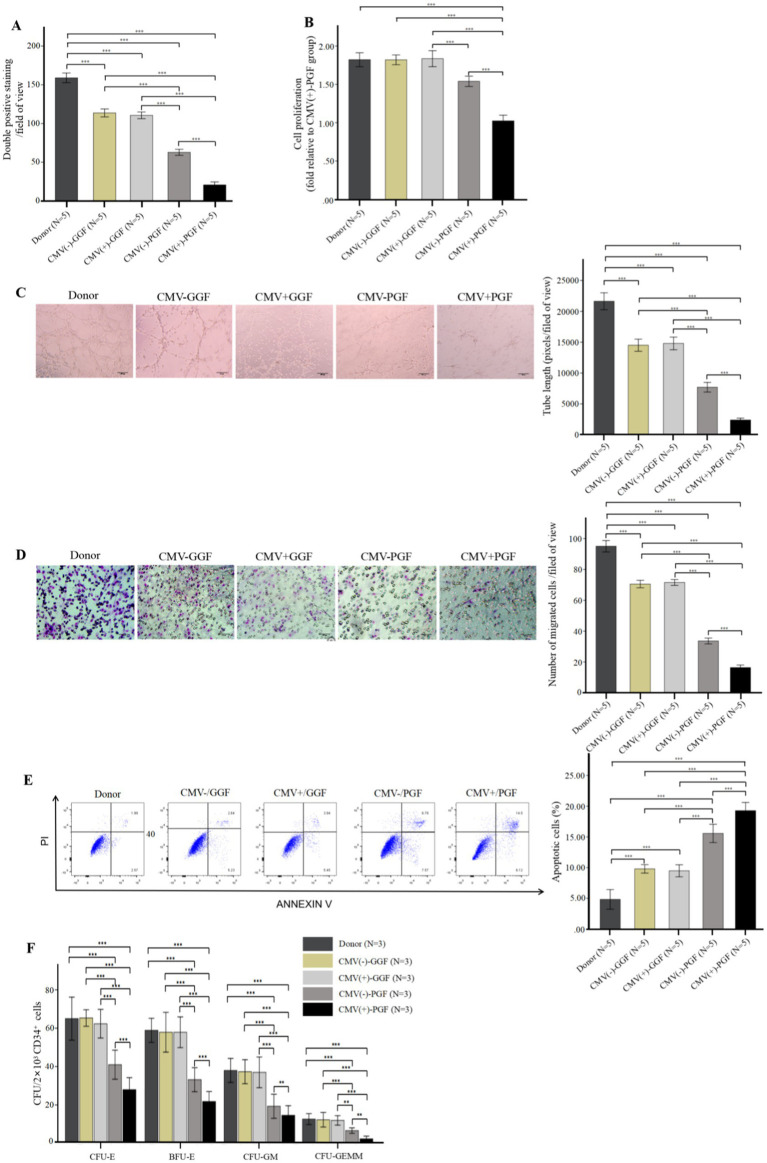
CMV impairs BM-EPCs functions in patients with CMV-emia after allo-HSCT. The functions of cultivated BM-EPCs at day 7 in culture in donor group, CMV(−) + GGF group, CMV(+) + GGF group, CMV(−) + PGF group and CMV(+) + PGF group were detected through double positive staining cells **(A)**, cell proliferation **(B)**, tube formation **(C)**, migration **(D)**, apoptosis **(E)**, hematopoietic support (CFU plating efficiency of CD34+ BM cells cocultured with BM-EPCs) **(F)**. ***, *p* < 0.001; **, *p* < 0.01; *, *p* < 0.05. Scale bars, 200um, original magnification ×10. Data shown is for one representative experiment out of 5 biological replicates, significance determined by one-way ANOVA and *post-hoc* tests.

**Figure 3:**
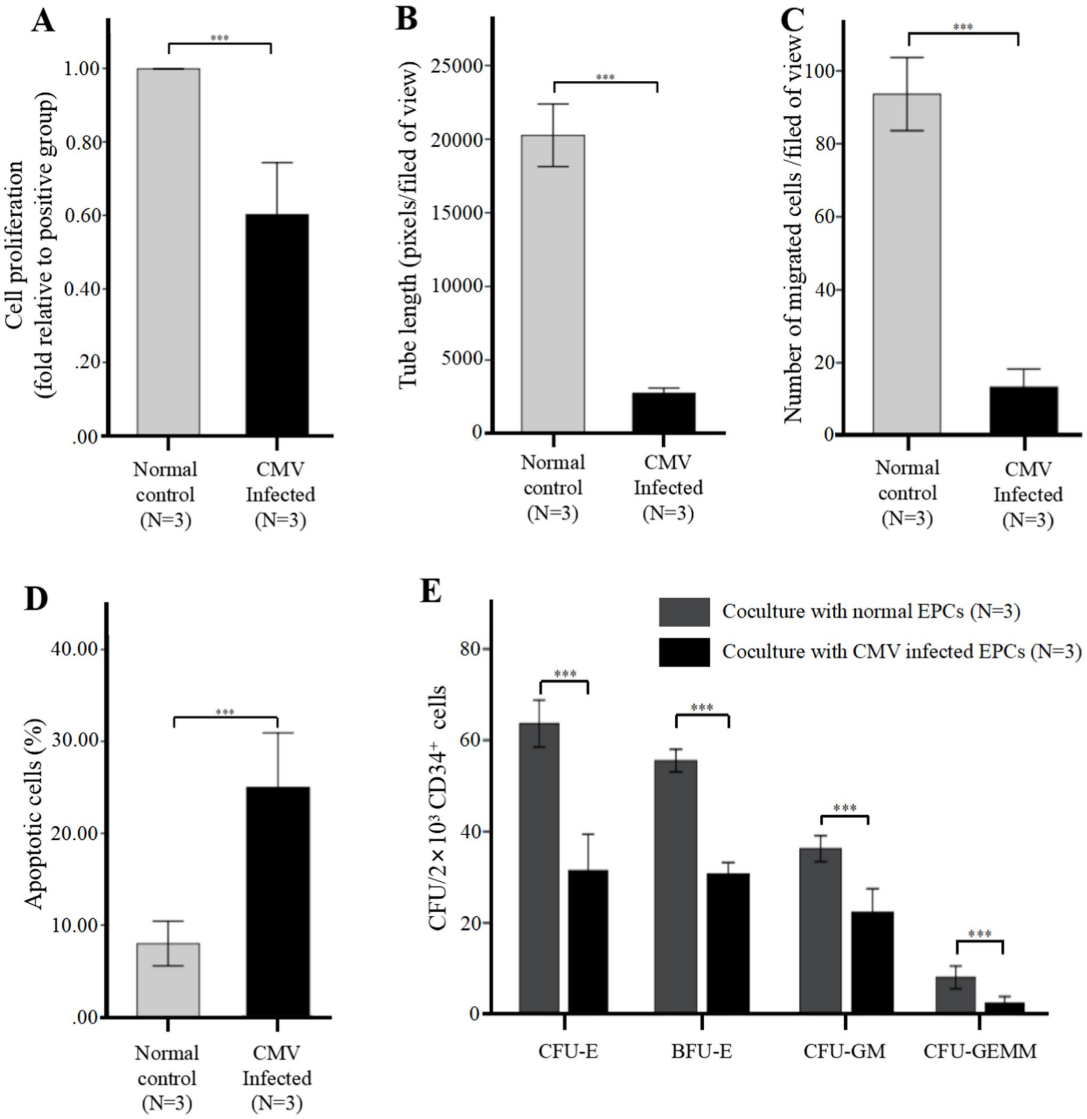
CMV impairs BM-EPCs functions *in vitro* infection experiments (after infection at 96 h). For infection *in vitro*, the adherent BM-EPCs from healthy donors of cultivation at 1–2 passage were exposed to HCMV AD169 at a multiplicity of infection (MOI) of 5 PFUs per cell for 2 h. Following infection, cells were washed in phosphate-buffered saline (PBS) to remove unabsorbed virus. Continuous cultures were established for 96 h, and BM-EPCs were collected for the following experiments. The adherent BM-EPCs from healthy donors of cultivation at 1–2 passage were exposed to HCMV AD169 (CMV infected group) and not exposed to HCMV AD169 (normal control group). Both groups were observed through an inverted microscope. Active CMV infection was characterized by expression of CMV pp65 protein (green) in the nuclear through an inverted fluorescent microscope (scale bars, 200um, original magnification ×10). **(A)** Cell proliferation of cultivated BM-EPCs at day 7 in culture among subjects in CMV infected group and normal control group. The functions of BM-EPCs in CMV infected group and normal control group were detected through tube formation **(B)**, migration **(C)**, apoptosis **(D)** and hematopoietic support (CFU plating efficiency of CD34+ BM cells cocultured with BM-EPCs) **(E)**. Significance determined by one-way ANOVA and *post-hoc* tests. ***, *p* < 0.001; **, *p* < 0.01; *, *p* < 0.05.

**Figure 4:**
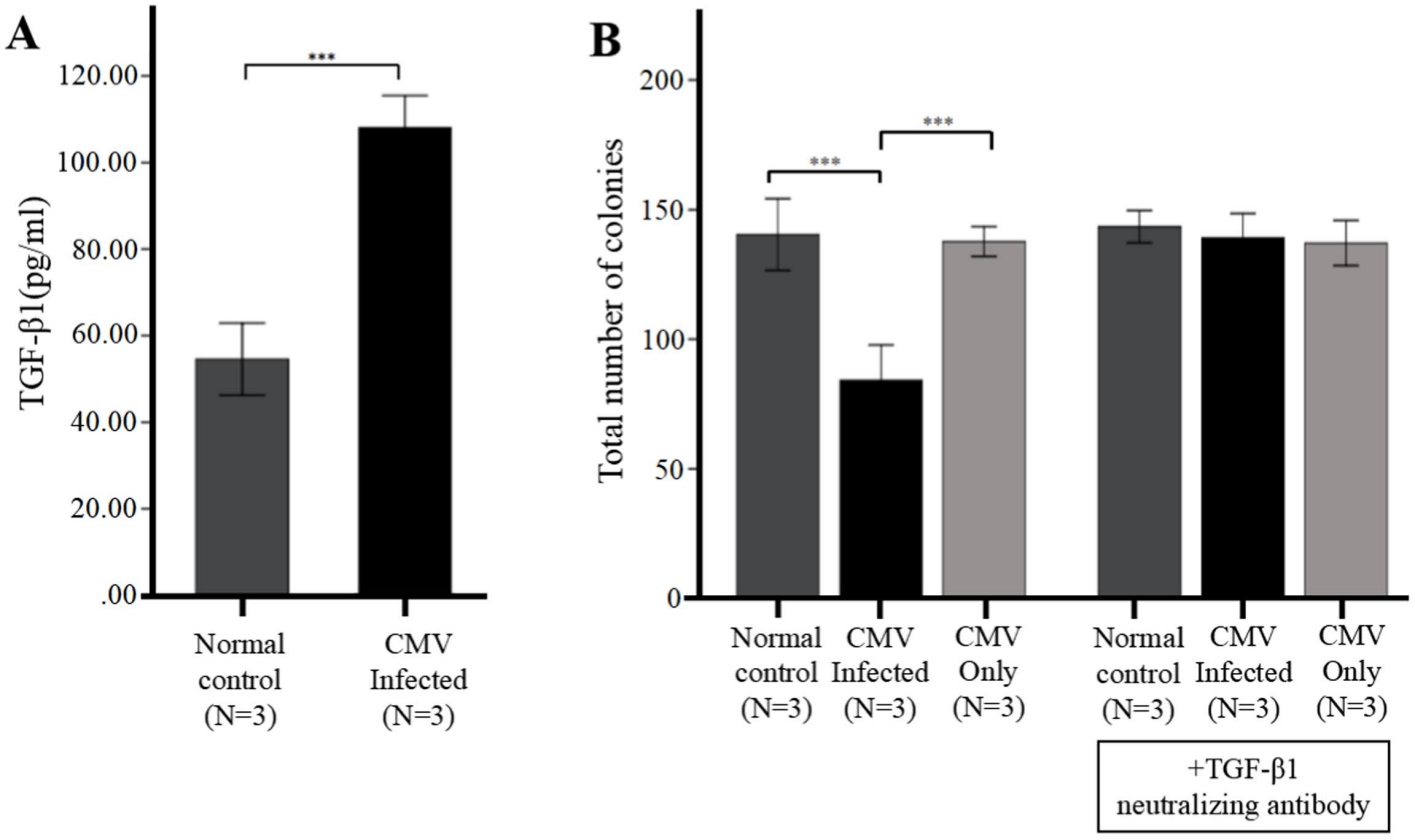
CMV directly suppresses CFU plating efficiency via upregulation of TGF-β1 from infected BM-EPCs. The adherent BM-EPCs from healthy donors of cultivation at 1–2 passage were exposed to HCMV AD169 at a MOI of 5 PFUs per cell for 2 h. Following infection, cells were washed in PBS to remove unabsorbed virus. Then the BM-EPCs with CMV infection (CMV Infected group) and without CMV infection (Normal control group) were plated in serum-free media for an additional 96 h prior to collection of cell-free supernatants (secretome samples). **(A)** Cell-free secretome samples from BM-EPCs in infected group or normal control group were analyzed for secreted TGF-β1 by ELISA. **(B)** To determine the effect of the CMV secretome from infected BM-EPCs, the CD34+ BM cells pretreated with or without anti-TGF-β antibody were mixed with secretome samples from BM-EPCs in CMV infected group and normal control group, plated at 2 × 10^3^ cells per dish in Methocult H4434 and cultured for 14 days. To detect the influence of CMV infecting CD34+ BM cells that led to impairment of CFU plating efficiency of CD34+ BM cells, CD34+ BM cells pretreated with or without anti-TGF-β antibody were also mixed with serum-free media containing the same copies of CMV as secretome samples from BM-EPCs in CMV infected group. Total colonies from a representative experiment are shown. Significance determined by one-way ANOVA and *post-hoc* tests. ***, *p* < 0.001; **, *p* < 0.01; *, *p* < 0.05.

**Figure 5:**
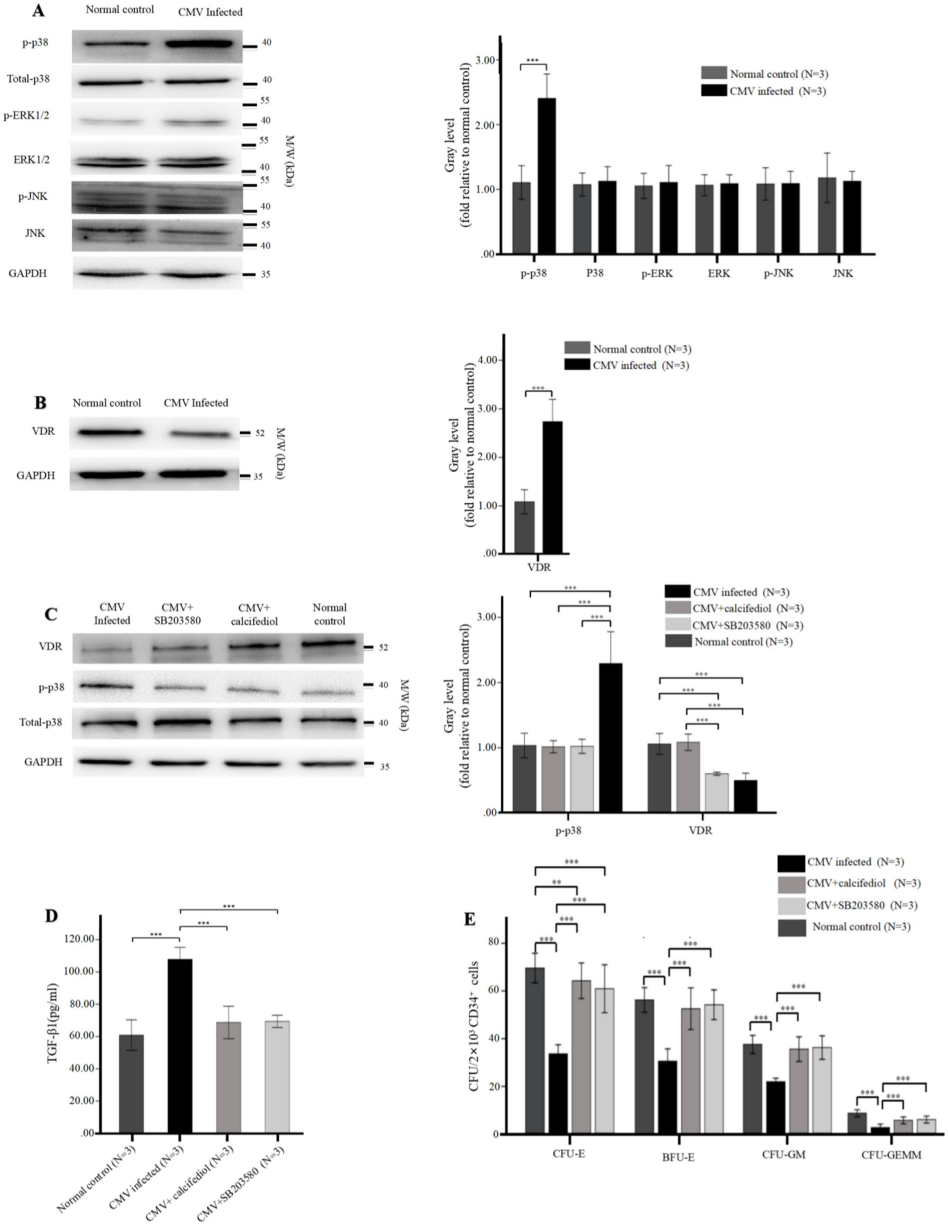
**(A,B)** Reduced levels of VDR and elevated intracellular phospho-p38 in BM-EPCs infected by CMV. The adherent BM-EPCs from healthy donors of cultivation at 1–2 passage were exposed to HCMV AD169 (CMV infected group) and not exposed to HCMV AD169 (normal control group) as described in Figure 2. **(A)** Representative western blots of MAPKs (phospho-p38, total p38, phospho-ERK, total ERK, phospho-JNK, total JNK) and GADPH in BM-EPCs from the CMV infected group and normal control group. **(B)** Representative western blots of VDR and GADPH in BM-EPCs from the CMV infected group and normal control group. Data are expressed as the fold of gray level relative to normal control (mean ± SEM). ***, *p* < 0.001; **, *p* < 0.01; *, *p* < 0.05. **(C–E)** The VDR agonist and p38 inhibitor similarly affected the secretion of TGF-β1 and hematopoietic support capability of BM-EPCs. The cultivated BM-EPCs from healthy donor were exposed to HCMV AD169 *in vitro* and then incubated with VDR agonist (calcitriol, 50 nM) or p38 inhibitor (SB203580, 10uM) for 24 h. **(C)** Representative western blots of VDR, phospho-p38, total p-38, and GADPH in BM-EPCs from the CMV infected group. Data are expressed as the fold of gray level relative to normal control (mean ± SEM). **(D)** BM-EPCs were infected with CMV, CMV + calcitriol and CMV + SB203580 as described above. Cell-free secretome samples from BM-EPCs in the above three groups were analyzed for secreted TGF-β1 by ELISA as described in Figure 3. **(E)** The CD34+ BM cells were mixed with diluted (1:5) secretome samples from BM-EPCs in CMV group, CMV + calcitriol group and CMV + SB203580 group, plated at 2 × 10^3^ cells per dish in Methocult H4434 and cultured for 14 days. Colonies from a representative experiment are shown. Data shown is for one representative experiment out of 3 biological replicates, significance determined by one-way ANOVA and *post-hoc* tests. ***, *p* < 0.001; **, *p* < 0.01; *, *p* < 0.05.

The data is reported as the average value plus or minus the standard deviation, as specified in the captions of Figures 1–5. Comparisons among the groups were conducted by employing 1-way ANOVA analysis and post-hoc tests of variance for statistical analyses. The analyses were conducted utilizing SPSS 25.0 for all statistical computations. The statistical significance was determined at a threshold of *p* values < 0.05.

## 3 Results

### 3.1 HCMV infects EPCs

The characterization of cultivated BM-EPCs was shown in (Supplementary Figure S2). To investigate the *in vivo* infection of BM-EPCs by HCMV in clinical patients, 7-day cultured BM-EPCs from five groups were examined for pp65 expression using IF: donor group, HCMV(−) + GGF group, HCMV(+) + GGF group, HCMV(−) + PGF group, and HCMV(+) + PGF group. The results demonstrated that pp65 expression was exclusively observed in primary BM-EPCs derived from patients in the HCMV(+) + PGF group (Figure 1A). Conversely, no detectable pp65 expression was found in BM-EPCs obtained from the remaining four groups.

*In vitro* infection of BM-EPCs with AD169 (HCMV Infected group) resulted in a substantial proportion of cells (>90%) exhibiting pp65 expression, while non-infected BM-EPCs showed no detectable pp65 expression (Normal control group). (Figure 1B).

### 3.2 HCMV impairs the function of BM-EPCs

#### 3.2.1 HCMV effects on proliferation, angiogenesis, and apoptosis of BM-EPCs

In BM-EPCs derived from clinical patients and cultured for a duration of 7 days, the quantity of cells exhibiting double-positive staining (Figure 2A), cellular proliferation(Figure 2B), capacity for tube formation (Figure 2C), and capacity for migration (Figure 2D) were significantly lower in the HCMV(+) + PGF group compared to the other four groups, including donor, HCMV(−) + GGF, HCMV(+) + GGF and HCMV(−) + PGF group. Additionally, the apoptosis level of were significantly lower in the HCMV(+) + PGF group compared to the other four groups (Figure 2E).

Further evaluation of BM-EPCs function in infection experiments by CMV *in vitro* showed that BM-EPCs in HCMV Infected group had significantly decreased the proliferation rate (Figure 3A), tube formation (Figure 3B) and migration (Figure 3C) compared with BM-EPCs in Normal control group. Additionally, infected BM-EPCs exhibited an elevated level of apoptosis (Figure 3D).

#### 3.2.2 HCMV effects on hematopoietic support capacity of BM-EPCs

Hematopoietic support capacity of BM-EPCs was determined by efficiency of CFU plating for CD34+ BM cells following their co-culture with BM-EPCs. In BM-EPCs derived from clinical patients and cultured for a duration of 7 days, the co-cultured CD34+ BM cells in the HCMV(+) + PGF group exhibited significantly lower CFU plating efficiency, as determined by decreased numbers of CFU-E, BFU-E, CFU-GM, and CFU-GEMM, compared to those in the other four groups. (Figure 2F).

After *in vitro* CMV infection, the co-cultured CD34+ BM cells in the HCMV Infected group exhibited significantly lower CFU plating efficiency compared to those in the Normal control group, as determined by decreased numbers of CFU-E, BFU-E, CFU-GM, and CFU-GEMM. (Figure 3E).

### 3.3 HCMV suppresses hematopoietic support capacity via secretion of TGF-β1

The expression of hematopoietic-related cytokines in BM-EPCs from clinical patients was assessed using qRT-PCR. The results of our study revealed a significant difference solely in the transcription of TGF-β1 among the aforementioned five groups. The transcription level of TGF-β1 in BM-EPCs from the HCMV(+) + PGF group exhibited the highest expression compared to the other four groups (Table 1). However, no significant difference was observed among the donor, HCMV(−) + GGF, HCMV(+) + GGF, and HCMV(−) + PGF groups.

**Table 1:** Transcription cytokines of primary bone marrow BM-EPCs in patients.

Gene	2-△△ct					*P*
	Donor (*N* = 3)	CMV(−) + GGF (*N* = 3)	CMV(+) + GGF (*N* = 3)	CMV(−) + PGF (*N* = 3)	CMV(+) + PGF (*N* = 3)	
G-CSF	0.98 ± 0.03^a^	1.02 ± 0.07^a^	1.00 ± 0.03^a^	1.04 ± 0.09^a^	0.95 ± 0.06^a^	0.514
GM-CSF	1.04 ± 0.08^a^	1.01 ± 0.04^a^	1.01 ± 0.04^a^	1.03 ± 0.06^a^	0.96 ± 0.01^a^	0.428
IL-6	0.99 ± 0.02^a^	1.00 ± 0.06^a^	1.05 ± 0.04^a^	1.07 ± 0.07^a^	1.11 ± 0.14^a^	0.366
TPO	1.01 ± 0.06^a^	1.03 ± 0.09^a^	1.01 ± 0.04^a^	0.98 ± 0.05^a^	1.01 ± 0.01^a^	0.931
SCF	1.06 ± 0.12^a^	0.95 ± 0.02^a^	1.04 ± 0.05^a^	1.02 ± 0.13^a^	0.97 ± 0.04^a^	0.527
SDF-1	1.09 ± 0.12^a^	1.02 ± 0.14^a^	1.00 ± 0.05^a^	1.12 ± 0.20^a^	0.95 ± 0.06^a^	0.240
TGF-β1	1.16 ± 0.09^a^	1.03 ± 0.09^a^	0.99 ± 0.01^a^	1.12 ± 0.15^a^	**2.71 ± 0.15** ^**b**^	**<0.001**

BM-EPCs, endothelial progenitor cells; CMV, cytomegalovirus; PGF, poor graft function; GGF, good graft function; G-CSF, granulocyte colony-stimulating factor; GM-CSF, granulocyte-macrophage colony-stimulating factor; IL-6, interleukin-6; TPO, thrombopoietin; SCF, stem cell factor; SDF-1, stromal cell-derived factor-1; TGF-β1, transforming growth factor beta 1. Significance determined by one-way ANOVA and *post-hoc* tests. Superscript: on the same line, the same letters in the top corner indicate no significant difference between groups; different letters in the top corner indicate a significant difference between groups. Bold values, a significant difference between groups (*P* < 0.05).

*In vitro* infection experiments, we further identified the HCMV-induced secretion of TGF-β1 by ELISA. The data presented in Figure 4A demonstrated that secretion of TGF-β1 in BM-EPCs could be induced by HCMV. Supernatants were added to healthy donor derived CD34+ BM cells to quantitate hematopoietic colony formation. The data presented in Figure 4B demonstrated that under identical culture conditions, the supernatants obtained from the HCMV Infected group exhibited a suppressive effect on colony formation compared to those obtained from the Normal control group. To detect the direct influence of HCMV on CD34+ BM cells that led to impairment of CFU plating efficiency of CD34+ BM cells, CD34+ BM cells were also mixed with serum-free media containing the same copies of HCMV as secretome samples from BM-EPCs in HCMV infected group (HCMV only group). The result showed that no significant reduction CFU plating efficiency of CD34+ BM cells was found in HCMV only group (Figure 4B).

To elucidate the potential impact of HCMV-induced TGF-β1 on hematopoietic colony formation inhibition, cell-free supernatants obtained from HCMV Infected group and Normal control group was supplemented with a neutralizing antibody against TGF-β1 at a concentration of 1 μg/mL. The data presented in Figure 4B demonstrated that after the introduction of a neutralizing antibody against TGF-β1, the levels of total hematopoietic colony formation in the HCMV-Infected group were restored to those observed in the Normal control group.

### 3.4 Phospho-p38 MAPK was elevated while VDR was reduced in BM-EPCs with HCMV infection

To investigate the impact of signaling pathways on impaired BM-EPCs *in vitro*, the protein levels of MAPKs (p38, ERK, JNK) and VDR in BM-EPCs were detected by western blot. After infected by HCMV *in vitro*, BM-EPCs infected by HCMV displayed higher level of phospho-p38 than normal control (Figure 5A). In contrast, total p38, total ERK, total JNK, and phospho-JNK levels did not exhibit a significant disparity between the BM-EPCs with and without HCMV infection (Figure 5A). In addition, significantly decreased levels of VDR expression were observed in BM-EPCs with HCMV infection (Figure 5B) than those without HCMV infection.

### 3.5 VDR regulates the activation of MAPK signaling pathway

To further investigate the interaction between p38 and VDR, we subsequently assessed the impact of VDR activation or p38 MAPK suppression by employing the VDR agonist (calcitriol) or p38 inhibitor (SB203580) on expression of p38 MAPK and VDR. The western blot analysis revealed that SB203580 effectively suppressed the phosphorylation of p38, while calcitriol significantly enhanced the expression of VDR (Figure 5C). Additionally, the expression of phospho-p38 was significantly downregulated in HCMV-infected EPCs upon treatment with either SB203580 or the calcitriol, while addition of SB203580 had no significant effect on expression of VDR (Figure 5C).

### 3.6 p38 inhibitor and VDR agonist similarly affected the secretion of TGF-β1 of BM-EPCs with HCMV infection *in vitro*

We investigated the impact of SB203580 inhibition or VDR activation on the functions of infected EPCs *in vitro*. In HCMV-infected EPCs, the addition of SB203580 or calcitriol resulted in a significant reduction in TGF-β1 secretion (Figure 5D) and an enhanced CFU plating efficiency in co-cultured CD34+ BM cells (Figure 5E) determined by increased number of CFU-E, BFU-E, CFU-GM, and CFU-GEMM. SB203580 and calcitriol similarly affected the secretion of TGF-β1 and hemopoietic support capacity of BM-EPCs with HCMV infection (Figures 5D,E).

## 4 Discussion

Our current study demonstrates that HCMV could infect EPCs, resulting in the functional impairments of EPCs. The suppression of VDR and its subsequent activation of thep38 MAPK signaling pathway, induced by HCMV infection, plays a vital role in regulating the secretion of TGF-β1 in BM-EPCs.

Emerging evidence demonstrates that the EPCs abnormality is associated with PGF development post-transplantation (Shi et al., 2016), but the mechanism of EPCs impairments in PGF patients is not clear. Clinical data indicates that HCMV infection is an important risk factor for PGF (Nakamae et al., 2011; Lv et al., 2021). However, no previous study has focued on the impact of CMV on BM-EPCs.

The dysfunction of BM-EPCs in patients with PGF was proven by Shi et al., which included changes in their ability to proliferate, migrate, resist apoptosis, and promote angiogenesis (Shi et al., 2016). Our findings further substantiated the presence of compromised functionality in BM-EPCs following HCMV infection, consistent with the aforementioned dysfunctional BM-EPCs in PGF. Notably, our results showed that the dysfunction observed in BM-EPCs derived from patients with HCMV-emia accompanied by PGF were more pronounced compared to those derived from patients without HCMV-emia accompanied by PGF, indicating the pivotal role of HCMV in the pathogenesis of BM-EPCs injury.

The process of hematopoiesis is precisely regulated through the action of hematopoietic cytokines (Giudice et al., 2021; Oltova et al., 2018). Our study revealed that HCMV-infected BM-EPCs exhibited excessive secretion of TGF-β1, a kind of hematopoietic inhibitory factor, which effectively impedes the growth and proliferation of CD34+ hematopoietic progenitor cells (HPCs) (Hancock et al., 2020; Blank and Karlsson, 2015; Bataller et al., 2019). Moreover, we observed that the introduction of a neutralizing antibody against TGF-β1 effectively reversed this suppression of hematopoiesis caused by HCMV infection, demonstrating that the crucial role of TGF-β1 in mediating the suppressive effects on hematopoiesis induced by HCMV.

The precise mechanisms underlying the dysfunction of EPCs remain incompletely elucidated. Shi et al. (2016) demonstrated that the dysfunction of BM-EPCs observed in patients with PGF is closely associated with the activation of p38 MAPK, emphasizing the crucial role of MAPKs in the pathogenesis of PGF. The study conducted by Wu-Wong et al. (2010) demonstrated that activation of the VDR enhances endothelial function in patients with chronic kidney disease. The levels of VDR were generally observed to be decreased in the blood cells of patients undergoing active HCMV infection following hematopoietic stem-cell transplantation (Robak et al., 2021). We provide experimental evidence that the expression of VDR on BM-EPCs was significantly reduced in the presence of CMV infection. Moreover, activation of VDR by calcitriol could effectively inhibit the secretion of TGF-β1 in CMV-infected BM-EPCs through the downregulation of the p38 MAPK pathway, thereby enhancing their hematopoietic support function. Therefore, targeting VDR and p38 MAPK using calcitriol May represent a promising therapeutic approach for augmenting the bone marrow microenvironment in individuals experiencing PGF due to CMV infection.

However, we acknowledge that the etiology of PGF displays significant heterogeneity. It would be intriguing and enlightening to conduct additional research on whether CMV-induced dysfunction of BM EPCs has a direct impact on hematopoiesis or if it influences the occurrence of PGF through its immunoregulatory properties. Further validation through *in vivo* experiments is required. Our preliminary data indicate that VDR agonist such as calcitriol represents a promising therapeutic approach for PGF by repairing impaired BM EPCs in patients with CMV viremia. However, further large-scale randomized clinical trials are warranted to substantiate our findings in the future.

In summary, HCMV could infect BM-EPCs and lead to their dysfunction. CMV enhances TGF-β1 secretion of BM-EPCs by activating p38 MAPK via a VDR-dependent mechanism, ultimately resulting in impaired hematopoietic progenitor support by BM EPCs. Our observations might make a substantial contribution to the pathogenesis of PGF after allo-HSCT, providing innovative therapeutic strategies targeting PGF.

## Data Availability

The original contributions presented in the study are included in the article/Supplementary material, further inquiries can be directed to the corresponding authors.
