# Supplementary material for: Cytomegalovirus results in poor graft function via bone marrow-derived endothelial progenitor cells
Source: Front Microbiol. 2024 Sep 18;15:1463335. doi: 10.3389/fmicb.2024.1463335 (PMC11445044; doi:10.3389/fmicb.2024.1463335)
Supplement: Supplementary file 1 [file Data_Sheet_1.pdf]

Table S1. Primers used in qRT-PCR

Name	Sequence	
G-CSF	Forward	ATAGCGGCCTTTTCCTCTACC
	Reverse	GCCATTCCCAGTTCTTCCAT
GM-CSF	Forward	ACCTGCCTACAGACCCGCC
	Reverse	GAAGTTTCCGGGGTTGGAGGGC
IL-6	Forward	GTAGCCGCCCCACACAGACAGCC
	Reverse	GCCATCTTTGGAAGGTTCAGG
TPO	Forward	GAATTGCTCCTCGTGGTCATGC
	Reverse	CAGTCTGCTGTGAAGGACATGG
SCF	Forward	CAGAGTCAGTGTCACAAAACCATT
	Reverse	TTGGCCTTCCTATTACTGCTACTG
SDF-1	Forward	CCCTTCAGATTGTAGCCCGG
	Reverse	CGATCCCAGATCAATGTGCC
TGF-β1	Forward	CCAACTATTGCATCAGCTCCA
	Reverse	TTATGCTGGTTGTACAGGG
INFγ	Forward	GCAGGTCATTCAGATGTAGCGG
	Reverse	TGTCTTCCTTGATGGTCTCCACAC
TNFα	Forward	TCTTCTCATTCCTGCTTGTG
	Reverse	ACTTGGTGGTTTGCTACG
TNFβ	Forward	CCTCACACCTTCAGCTGCCC
	Reverse	GAGAAACCATCCTGGAGGAA
GAPDH	Forward	GTCTCCTCTGACTTCAACAGCG
	Reverse	ACCACCCTGTTGCTGTAGCCAA
