# Supplementary material for: Cytomegalovirus results in poor graft function via bone marrow-derived endothelial progenitor cells
Source: Front Microbiol. 2024 Sep 18;15:1463335. doi: 10.3389/fmicb.2024.1463335 (PMC11445044; doi:10.3389/fmicb.2024.1463335)
Supplement: Supplementary file 2 [file Data_Sheet_2.pdf]

Table S2. Characteristics of allo-HSCT subjects

Characteristics	CMV(+)+PGF cases* (N=10)	CMV(-)+PGF cases* (N=10)	CMV(+)+GGF cases* (N=10)	CMV(-)+GGF cases* (N=10)	*P* value
**BM evaluated time (post-HSCT days)**	52.5(29-110)	52.5(30-114)	54.5(30-112)	52(31-114)	1.00
**Blood cell count**					
Median WBC (3×10^9/L) (range)	1.39(0.98-2.04)[a]	2.02(1.45-2.32)[b]	4.94(3.49-6.03)[c]	4.88(3.44-5.98)[c]	<0.0001
Median NEU (3×10^9/L) (range)	0.29(0.12-0.43)[a]	0.43(0.31-0.47)[b]	2.35(1.25-3.45)[c]	2.29(1.93-2.56)[c]	<0.0001
Median HGB (g/L) (range)	53(43-60)[a]	56(50-64)[b]	127(89-145)[c]	109.5(87-143)[c]	<0.0001
Median PLT (3×10^9/L) (range)	12(5-19)[a]	16(12-19)[b]	139(99-234)[c]	142.5(89-266)[c]	<0.0001
**Age at HSCT (years, median, range)**	27(19-39)	29(19-40)	28(18-40)	28(17-41)	0.96
**Sex (male/female)**	6/4	5/5	6/4	7/3	0. 93
**Underlying disease**					0. 89
AML	5	5	5	5	
ALL	4	4	4	5	
MDS	1	1	1	0	
**Transplanted cell dose**					
Transplanted total nucleated cell dose ($\times10^8$ /kg, median, range)	7.96(3.88-9.34)	7.16(5.02-9.51)	7.91(4.98-9.95)	7.32(5.04-10.00)	0. 96
Transplanted CD34+ total nucleated cell dose ($\times10^8$ /kg, median, range)	2.72(1.56-5.88)	2.69(2.04-5.55)	3.15(1.49-5.63)	2.76(1.72-5.84)	0. 59
**Donor source**					0. 84
HLA-identical sibling donor	6	5	6	6	
HLA–partially matched related donor	4	5	4	4	
**Sex mismatch**					0. 97
Male to female	1	2	2	3	
Male to male	5	4	5	4	
Female to male	1	2	1	2	
Female to female	3	2	2	1	
**ABO mismatch**					0. 73
No	6	6	5	3	
Major	2	1	2	4	
Minor	2	3	3	3	
**Pre-HSCT cycles of chemotherapy**	4(1-6)	3(1-5)	4(1-6)	3(2-5)	0. 66
**Conditioning**					0. 84
BU/CY	6	5	6	6	
BU/CY+ATG	4	5	4	4	
**History of aGVHD**	4	4	3	3	0. 29
**Onset of aGVHD (days, median, range)**	27(15-49)	31.5(17-45)	27(16-52)	21(17-46)	0. 72

**CMV IgG in donor and recipient before transplantation**					0. 98
Donor + vs recipient +	3	2	2	1	
Donor + vs recipient -	4	3	3	4	
Donor - vs recipient -	3	4	4	4	
Donor - vs recipient +	0	1	1	1	
**Copies of CMV peripheral blood at evaluate time**	8310(564-93928)	1861.5(678-43267)	—	—	0. 029

HSCT, Hematopoietic Stem Cell Transplantation; WBC, white blood cell count; NEU, neutrophil count; HGB, hemoglobin; PLT, platelet; AML, acute myelogenous leukemia; ALL, acute lymphocytic leukemia; MDS, myelodysplastic syndrome; ABO, ABO blood group; BU/CY, busulfan and cyclophosphamide; ATG, antithymocyte;globulin; aGVHD, acute GVHD; CMV, cytomegalovirus.

*Group matching criteria included age at HSCT (±2 years), pre-HSCT cycles of chemotherapy (±1 cycle), and BM evaluated time after HSCT (±5 day).

The continuous variables were compared using the Mann-Whitney U test or Kruskal-Wallis test, and the differences in frequency between the 2 groups were compared using the X2 test. The criterion for statistical significance was $P<0.05$.
