# Supplementary figures and images for: Cytomegalovirus results in poor graft function via bone marrow-derived endothelial progenitor cells
Source: Front Microbiol. 2024 Sep 18;15:1463335. doi: 10.3389/fmicb.2024.1463335 (PMC11445044; doi:10.3389/fmicb.2024.1463335)

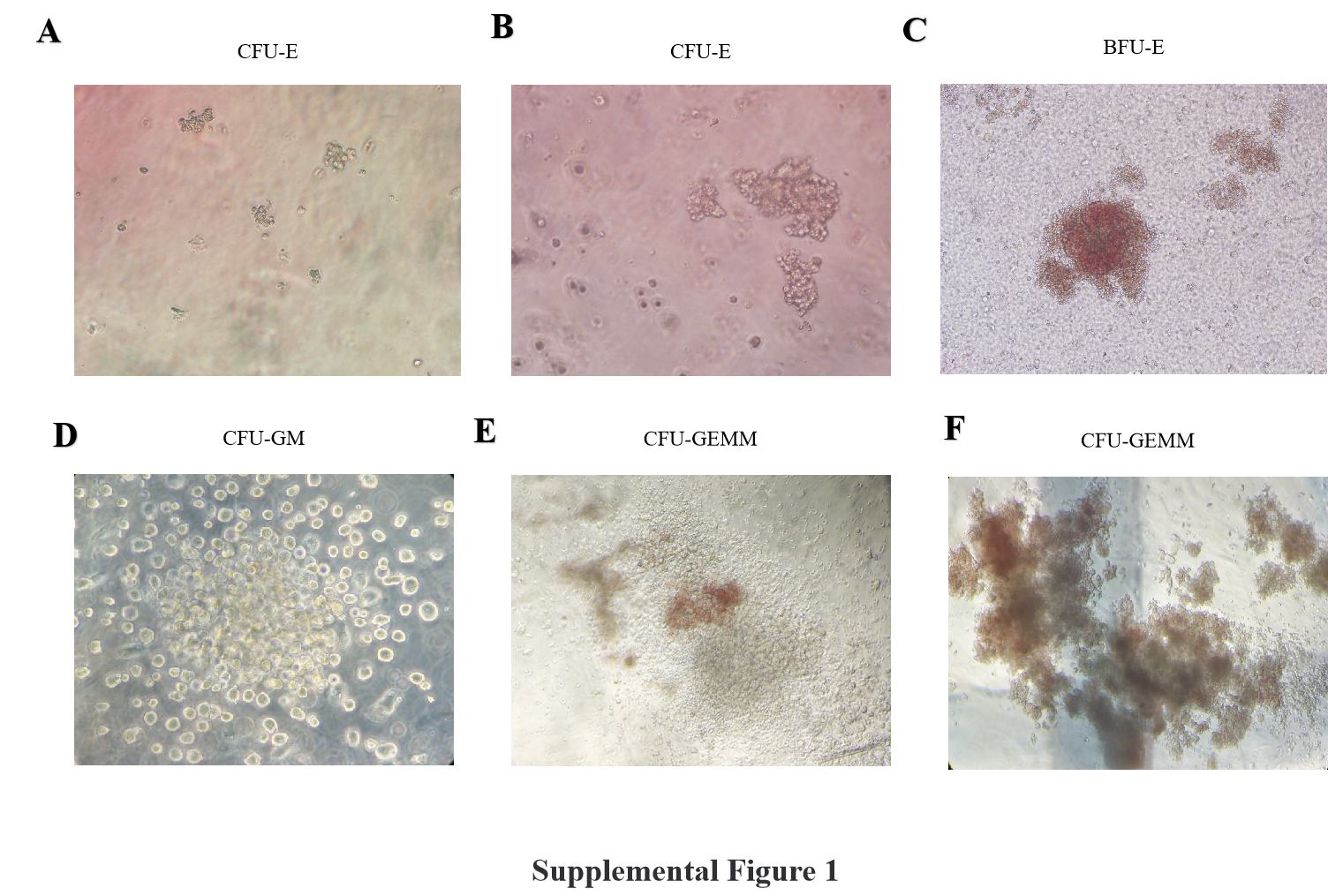

Supplement: Supplementary file 3 [file Image_1.PNG]

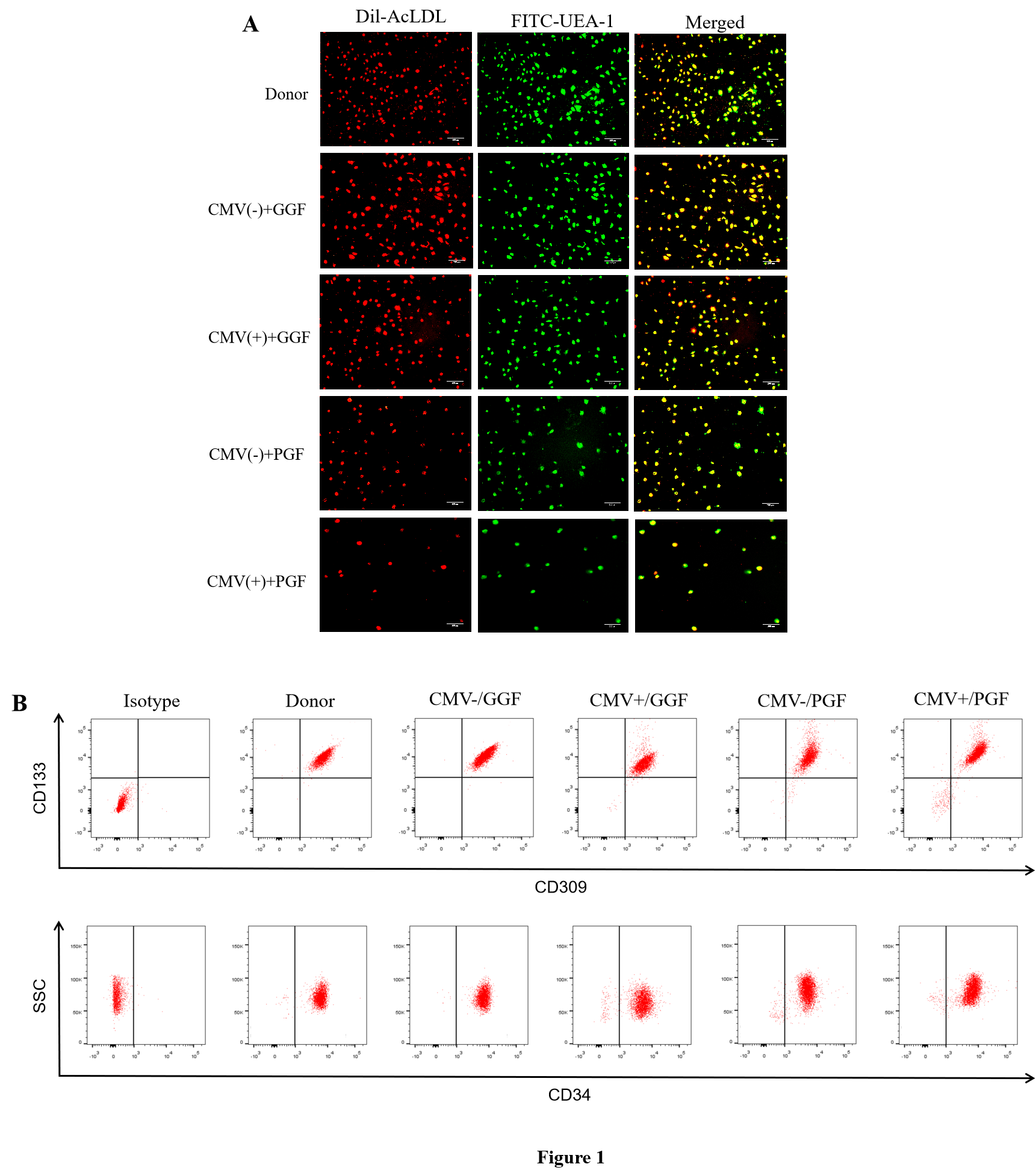

Supplement: Supplementary file 4 [file Image_2.PNG]
